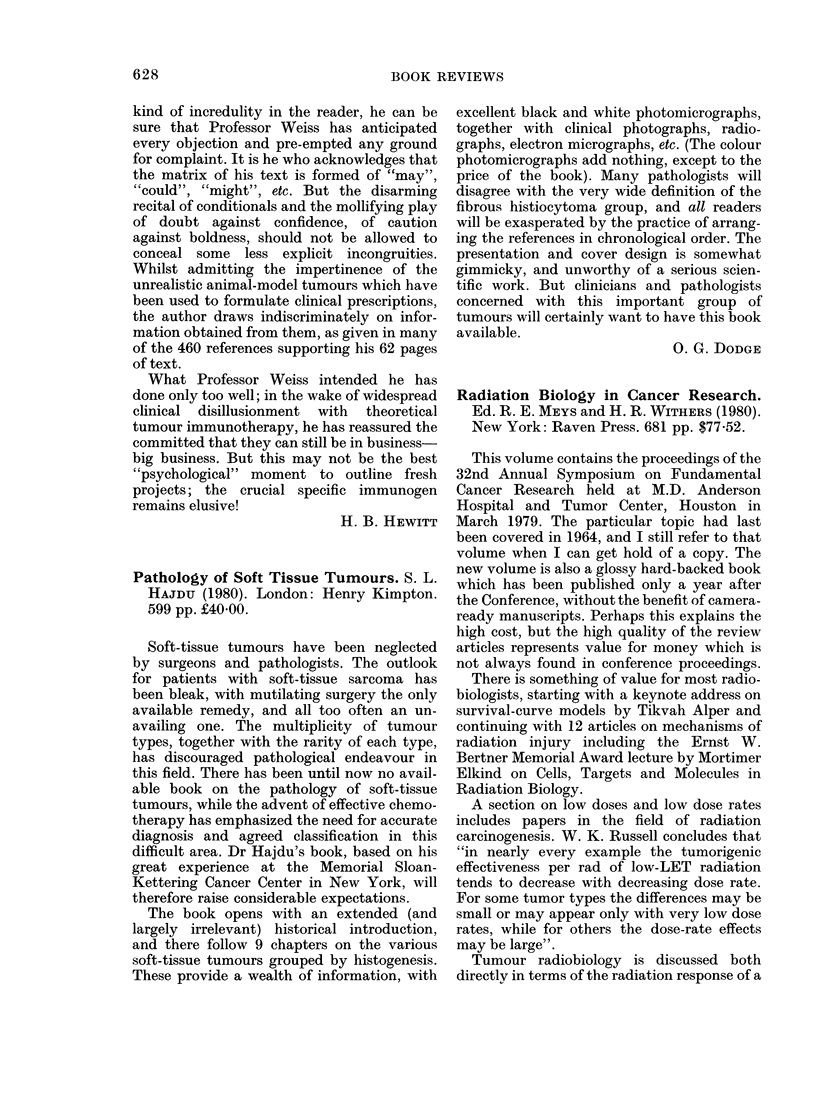# Pathology of Soft Tissue Tumours

**Published:** 1980-10

**Authors:** O. G. Dodge


**Pathology of Soft Tissue Tumours.** S. L. HAJDU (1980). London: Henry Kimpton. 599 pp. £40·00.

Soft-tissue tumours have been neglected by surgeons and pathologists. The outlook for patients with soft-tissue sarcoma has been bleak, with mutilating surgery the only available remedy, and all too often an unavailing one. The multiplicity of tumour types, together with the rarity of each type, has discouraged pathological endeavour in this field. There has been until now no available book on the pathology of soft-tissue tumours, while the advent of effective chemotherapy has emphasized the need for accurate diagnosis and agreed classification in this difficult area. Dr Hajdu's book, based on his great experience at the Memorial Sloan-Kettering Cancer Center in New York, will therefore raise considerable expectations.

The book opens with an extended (and largely irrelevant) historical introduction, and there follow 9 chapters on the various soft-tissue tumours grouped by histogenesis. These provide a wealth of information, with excellent black and white photomicrographs, together with clinical photographs, radiographs, electron micrographs, *etc.* (The colour photomicrographs add nothing, except to the price of the book). Many pathologists will disagree with the very wide definition of the fibrous histiocytoma group, and *all* readers will be exasperated by the practice of arranging the references in chronological order. The presentation and cover design is somewhat gimmicky, and unworthy of a serious scientific work. But clinicians and pathologists concerned with this important group of tumours will certainly want to have this book available.

O. G. DODGE